# Association between Yogurt Consumption and Intestinal Microbiota in Healthy Young Adults Differs by Host Gender

**DOI:** 10.3389/fmicb.2017.00847

**Published:** 2017-05-11

**Authors:** Yoshio Suzuki, Keiichi Ikeda, Kazuhiko Sakuma, Sachio Kawai, Keisuke Sawaki, Takashi Asahara, Takuya Takahashi, Hirokazu Tsuji, Koji Nomoto, Ravinder Nagpal, Chongxin Wang, Satoru Nagata, Yuichiro Yamashiro

**Affiliations:** ^1^Juntendo University School of Health and Sports ScienceChiba, Japan; ^2^Yakult Central InstituteTokyo, Japan; ^3^Probiotics Research Laboratory, Juntendo University Graduate School of MedicineTokyo, Japan; ^4^Department of Pediatrics, Tokyo Women’s Medical UniversityTokyo, Japan

**Keywords:** diet, gender, yogurt, probiotics, gut bacteria, *Lactobacillus*, RT-qPCR, short-chain fatty acids

## Abstract

Human intestinal microbiota are influenced by various factors viz. diet, environment, age, gender, geographical, and socioeconomic situation, etc. among which diet has the most profound impact. However, studies investigating this impact have mostly included subjects from diverse geographic/socioeconomic backgrounds and hence the precise effects of dietary factors on gut microbiota composition remain largely confounded. Herein, with an aim to evaluate the association between dietary habits, specifically yogurt consumption, and the gut microbiota in healthy young adults sharing similar age, lifestyle routine, geographical setting, etc., we conducted a cross-sectional study wherein 293 collegiate freshmen answered a questionnaire about their frequency of yogurt consumption over the last 2 months and provided stool specimens for microbiota analysis. Fecal microbiota were analyzed by highly sensitive reverse-transcription-quantitative-PCR assays targeting bacterial 16S rRNA molecules. Fecal organic acids were measured by HPLC. Overall, the gut microbiota were predominated (97.1 ± 8.6%) by *Clostridium coccoides* group, *Clostridium leptum* subgroup, *Bacteroides fragilis* group, *Bifidobacterium* and *Atopobium* cluster. Interestingly, after adjusting the data for yogurt consumption, females were found to have higher total bacterial (*P* = 0.013) and *Bifidobacterium* (*P* = 0.046) count and fecal pH (*P* = 0.007) and lower fecal concentration of total organic acids (*P* = 0.030), succinic acid (*P* = 0.007) and formic acid (*P* = 0.046) as compared to males. Altogether, yogurt consumption showed positive linear association with *Lactobacillus* and *Lactobacillus gasseri* subgroup in both male and female subjects; however, several gender-specific disparities were also detected in this yogurt-microbiota association. Yogurt consumption demonstrated a negative association with *L. sakei* subgroup, Enterobacteriaceae and *Staphylococcus* in males but shared a positive association with *L. casei* subgroup and succinic acid in female subjects. The study manifests the association between yogurt consumption and gut microbiota in a healthy homogeneous cohort and show how this association can differ by host gender. The findings should be helpful for prospective studies investigating the diet–microbiome interaction in human health and disease.

## Introduction

Human intestinal microbiota is composed of approximately 10^14^ bacterial cells, outnumbering our body’s own cells, and influences numerous aspects of our health and physiological condition. Various international research groups, including Human Microbiome Project (HMP), Metagenomics of the Human Intestinal Tract (MetaHIT), and others have demonstrated that the diversity of the human gut microbiota is influenced by various factors such as age, gender, country, residential area and diet (Mueller et al., 2006; Yatsunenko et al., 2012; Robles-Alonso and Guarner, 2014; Nakayama et al., 2015). Of these elements, diet plays a prominent and profound role in driving the intestinal microbiota composition wherein dietary habits can rapidly and reproducibly modulate the microbiota configuration (Sonnenburg and Bäckhed, 2016). We have now learnt that as our diet changed over evolutionary events, so did our intestinal microbiota (Ley et al., 2008). As a result, the interest of researchers in deciphering the effects of dietary factors on gut microbiome has progressively increased over the last years (David et al., 2014; Griffin et al., 2016). Concomitantly, a growing body of studies has also indicated multiple beneficial effects of yogurt consumption on human health, such as maintenance of a healthy gut microbiota, prevention of various gastrointestinal diseases etc. (Donovan and Shamir, 2014); however, the dynamics of the association between the frequency of yogurt consumption and the gut microbiota communities in people sharing same ethnicity, age, residential and socioeconomic environment remain unstudied. Interestingly, several studies have also reported gender-dependent differences in the human intestinal microbiota (Mueller et al., 2006; Bolnick et al., 2014); however, whether (and how) these differences pertain to the diet–microbiota association remain largely unclear. In this milieu, we herein conducted a cross-sectional study of a large cohort of healthy Japanese collegiate freshmen to (i) understand the association between the intestinal microbiota and the frequency of yogurt consumption in healthy young subjects and (ii) evaluate the role of host gender in this association. Notably, majority of the human microbiota studies have employed DNA-based sequencing/metagenomic methods (Mueller et al., 2006; Robles-Alonso and Guarner, 2014) which, while yielding important and comprehensive data on predominant bacterial clades, may not provide adequate information about the subdominant inhabitants (e.g., *Lactobacillus* subgroups and species) that are generally present in low numbers (e.g., <10^4-5^ cells/g feces) but still constitute a regular and indispensable component of our gut microbiota. To this end, we have previously developed a novel and sensitive analytical system for human fecal microbiota analysis based on reverse-transcription-quantitative-PCR (RT-qPCR) assays targeting bacterial rRNA molecules, wherein we have validated that this approach is relatively highly sensitive (∼100–1000 fold more sensitive than other molecular methods such as qPCR and t-RFLP) and provides fecal bacterial counts equivalent to those enumerated by culture and fluorescent-*in-situ*-hybridization methods (Matsuda et al., 2007; Kubota et al., 2010; Kurakawa et al., 2012, 2015a). Owing to these advantages, we employed this RT-qPCR approach in the present investigation so as to obtain the quantitative information on a wide-range of important predominant as well as subdominant gut bacterial inhabitants.

## Materials and Methods

### Study Design

The study included first-year graduate students (enrolled at Juntendo University) residing in the same dormitory. After 3 months of matriculation, all subjects (*n* = 435) were given a thorough explanation of the objectives, methods and ethical considerations of the study. Following the exclusion based on gastrointestinal symptoms or recent use of antibiotics, a total of 293 (67.3%) healthy students (212 male; 81 female) were finally selected for participation in the study. All subjects gave prior written informed consent for participation. The general characteristics of the subjects are summarized in **Table 1**.

**Table 1:** General characteristics of the study subjects.

	Male	Female
N	212	81
Age (y)	19.6 ± 0.7 (18–23)	19.7 ± 0.7 (19–22)
Height (cm)	172.0 ± 6 (158–194)	160.1 ± 5.4 (146–173)
Weight (kg)	64.5 ± 11.3 (48–165)	52.9 ± 5.8 (41–68)
BMI (kg/m^2^)	21.8 ± 3.1 (16.8–47.2)	20.6 ± 1.8 (17.3–25.6)

*N, number of subjects. Data are presented as Mean ± SD (minimum – maximum).*

All the students had enrolled at the university and moved in the dormitory during early April (2011), and the study survey began in mid-June (2011). Hence, it had already been nearly 3 months since the students have experienced a major change in their life circumstances and routine.

Subjects were asked to provide fecal specimen and answer a questionnaire about their habitual frequency of yogurt/ prebiotics (hereafter collectively referred to as yogurt) consumption over the last 2 months by selecting one of the following four options: <1, 1–2, 3–5, or 6–7 days in a week (d/wk). Every option was chosen by at least more than 8% of the subjects, male as well as female, although the distribution differed significantly (*p* < 0.001) (Supplementary Table 1).

### Ethics Statement

The study was conducted in accordance with the ‘Declaration of Helsinki’ guidelines. All the procedures involving human subjects were approved by the Ethics Committee of Juntendo University School of Health and Sports Science (Approval number: #23-12).

### Sample Collection

Following the obtainment of informed consent, the subjects were provided with a fecal sample collection kit along with the instructions for sample collection. Subjects collected freshly voided fecal samples (≈0.5 g) into the fecal collection tube (Sarstedt AG & Co., Numbrecht, Germany) containing 2 ml RNA*later* (an RNA stabilization solution; Ambion, Austin, TX) (for microbiota analysis) and an empty tube (for organic acid analysis and pH measurement). Samples were placed immediately in a cooling box containing refrigerants and were transported (within 24 h) to the Yakult Central Institute where these were stored immediately after collection at 4°C (tubes with RNA*later*) and -20°C (tubes without RNA*later*) in a Bio-safety Level II laboratory until further processing.

### Bacterial Quantification

To quantify the major gut bacterial groups, the samples were subjected a primary treatment (homogenization) step and the total RNA extraction as per the previously described method (Matsuda et al., 2007, 2009; Kubota et al., 2010). The fecal count of major gut bacterial groups were assessed by using a sensitive RT-qPCR analytical system based on the quantification of bacterial 16S or 23S rRNA molecules (Matsuda et al., 2007, 2009; Kubota et al., 2010; Tsuji et al., 2012; Nagpal et al., 2016). Three serial dilutions of the extracted RNA sample were used for RT-qPCR (Matsuda et al., 2007, 2009; Kubota et al., 2010; Tsuji et al., 2012), and the threshold cycle (C_t_) values in the linear range of the assay were applied to the standard curve to obtain the corresponding bacterial cell count in each nucleic acid sample. These data were then used to determine the number of bacteria per sample. The details of the analytical specificity and sensitivity of the RT-qPCR assays using various group-, genus-, and species-specific primer sets have been described previously (Matsuda et al., 2009). Briefly, the minimum detection limit (log_10_ cells/g feces) for various bacterial clades analyzed in here were as follows: 5.0 for *Clostridium coccoides* group, *Clostridium leptum* subgroup, *Bacteroides fragilis* group, *Bifidobacterium, Atopobium* cluster, and *Prevotella*; 2.4 for *Clostridium perfringens, Lactobacillus, Lactobacillus gasseri* subgroup, *L. reuteri* subgroup, *L. ruminis* subgroup, *L. plantarum* subgroup, *L. sakei* subgroup, and *L. brevis*; 3.1 for *L. casei* subgroup and *Staphylococcus*; 4.0 for *L. fermentum* and *Enterococcus*; and 4.1 for Enterobacteriaceae. The total bacterial count was estimated as the sum of counts of all of the abovementioned bacteria. The count of *Lactobacillus* was expressed as the sum of the counts of six subgroups (*L. casei* subgroup, *L. gasseri* subgroup, *L. plantarum* subgroup, *L. reuteri* subgroup, *L. ruminis* subgroup, and *L. sakei* subgroup) and two species (*L. brevis* and *L. fermentum*).

### Measurement of Fecal Organic Acids and pH

The fecal concentration of organic acids was determined by using methods described previously (Tsuji et al., 2012). Briefly, the frozen sample was homogenized in four volumes of 0.15 mol/L perchloric acid and allowed to stand at 4°C for 12 h. The suspension was centrifuged at 20,400 × *g* at 4°C for 10 min. Then, the resulting supernatant was passed through a filter with a pore size of 0.45 μm (Millipore Japan, Tokyo, Japan). The sample was analyzed for organic acids using a high-performance liquid chromatography (HPLC) system (432 Conductivity Detector; Waters Co., Milford, MA, USA). Detection limits of organic acids (μmol/g feces) were as follows: succinic acid, 0.075; lactic acid, 0.2; formic acid, 0.05; acetic acid, 0.4; propionic acid, 0.5; butyric acid, 0.55; and iso-valeric acid, 0.8. Fecal pH was measured by directly inserting the IQ 150 pH/Thermometer (IQ Scientific Instruments, Inc., Carlsbad, CA, USA) into the fecal sample.

### Statistical Analyses

Fecal bacterial counts (log_10_ cells/g feces) and organic acid concentrations (μmol/g feces) are expressed as mean ± standard deviation or marginal estimated mean ± standard error. We used non-parametric statistical assessment because the normality of distribution was not hypothesized for most of the parameters including questionnaire answers, and fecal bacterial counts and organic acid concentrations. For the statistical calculation of fecal bacterial count and organic acids concentration, a value of half of the detection limit was assigned in case the count or concentration was below the detection limit. Mean difference of two independent and paired groups was analyzed by Mann–Whitney’s *U*-test. Gender differences in fecal microbiota and organic acids were analyzed by generalized linear model (GLM) controlled for frequency of yogurt consumption. The parameter settings were as follows: scale response: linear; factor: gender; and covariates: yogurt frequency. The relation between frequency of yogurt consumption and fecal microbiota and organic acids was analyzed by Jonckheere–Terpstra’s trend test. The linearity of microbiota and organic acids with yogurt frequency was analyzed by GLM using yogurt frequency as covariate in data stratified by gender. When linearity was hypothesized (*P* < 0.05), marginal estimated means were calculated and compared by GLM using yogurt frequency as factor. In order to compare the mean difference between gender in subjects taking yogurt less than once in a week, marginal estimated means were calculated and compared by GLM using total un-stratified data with gender and yogurt frequency as factors. The statistical analyses were performed using IBM SPSS Statistics ver. 19 (IBM Japan, Tokyo, Japan). Values of *P* < 0.05 were considered statistically significant.

## Results

### Overall Profiles of Fecal Microbiota and Organic Acids

In this cohort of healthy collegiate freshmen, the fecal microbiota were found to be composed predominantly of *C. coccoides* group, *C. leptum* subgroup, *B. fragilis* group, *Bifidobacterium* and *Atopobium* cluster, which collectively constituted 97.1 ± 8.6% of the total bacterial count (10.65 ± 0.36 log_10_ cells/g feces). The counts of all of these five bacterial groups shared positive correlation with total bacterial count (*P* < 0.001); *C. coccoides* group (*R* = 0.668), *C. leptum* subgroup (*R* = 0.734), *B. fragilis* group (*R* = 0.564), *Bifidobacterium* (*R* = 0.641) and *Atopobium* cluster (*R* = 0.544). In contrast, *Prevotella* was detected (>10^5^ cells/g feces) only in 28.7% of the subjects (**Table 2**). Enterobacteriaceae, *Lactobacillus* and *Enterococcus* were the next abundant bacterial groups in the fecal microbiota (**Table 2**). Among lactobacilli, *L. gasseri* subgroup was the most predominant clade (**Table 2**). *C. perfringens* and *Staphylococcus* were detected (>2.4 and >3.1 log_10_ cells/g feces, respectively) in 38.2 and 52.6% of the subjects, respectively (**Table 2**). Acetic, propionic and butyric acids were the major components of fecal organic acids (83.10 ± 38.89 μmol/g feces). The pH of feces was 6.44 ± 0.59 (**Table 2**).

**Table 2:** Fecal microbiota, organic acids and pH in healthy Japanese young adults (*n* = 293).

	Mean ±*SD*	Prevalence (%)
Microbiota (log_10_ cells/g feces)		
Total bacteria	10.6 ± 0.4	100.0
*C. coccoides* group	10.0 ± 0.4	100.0
*C. leptum* subgroup	9.7 ± 0.7	100.0
*B. fragilis* group	9.7 ± 1.0	99.0
*Bifidobacterium*	9.5 ± 1.7	96.9
*Atopobium* cluster	8.9 ± 1.1	98.6
*Prevotella*	4.0 ± 2.6	28.7
*C. perfringens*	2.7 ± 2.0	38.2
*Lactobacillus*	5.4 ± 1.6	93.9
*L. gasseri* subgroup	4.5 ± 2.0	77.8
*L. reuteri* subgroup	2.6 ± 1.7	45.4
*L. ruminis* subgroup	2.2 ± 1.9	24.6
*L. plantarum* subgroup	2.5 ± 1.5	48.5
*L. sakei* subgroup	2.2 ± 1.5	37.2
*L. casei* subgroup	2.8 ± 1.8	34.8
*L. brevis*	1.4 ± 0.7	7.8
*L. fermentum*	2.6 ± 1.4	18.1
Enterobacteriaceae	6.6 ± 1.6	91.5
*Staphylococcus*	3.4 ± 1.9	52.6
*Enterococcus*	5.4 ± 1.9	80.9
Organic acids (μmol/g feces)		
Total organic acids	83.1 ± 38.9	100.0
Succinic acid	3.9 ± 9.4	31.1
Lactic acid	0.6 ± 4.1	5.1
Formic acid	0.4 ± 1.4	10.6
Acetic acid	52.8 ± 25.4	100.0
Propionic acid	15.9 ± 9.3	98.0
Butyric acid	9.5 ± 8.3	92.5
Isovaleric acid	0.5 ± 0.6	1.4
Fecal pH	6.4 ± 0.6	-

*Prevalence (detection rate, %) is expressed as the percentage of subjects in which the specific bacterium/organic acid was detected.*

### Gender-Specific Differences in Fecal Microbiota and Organic Acids

**FIGURE 1:**
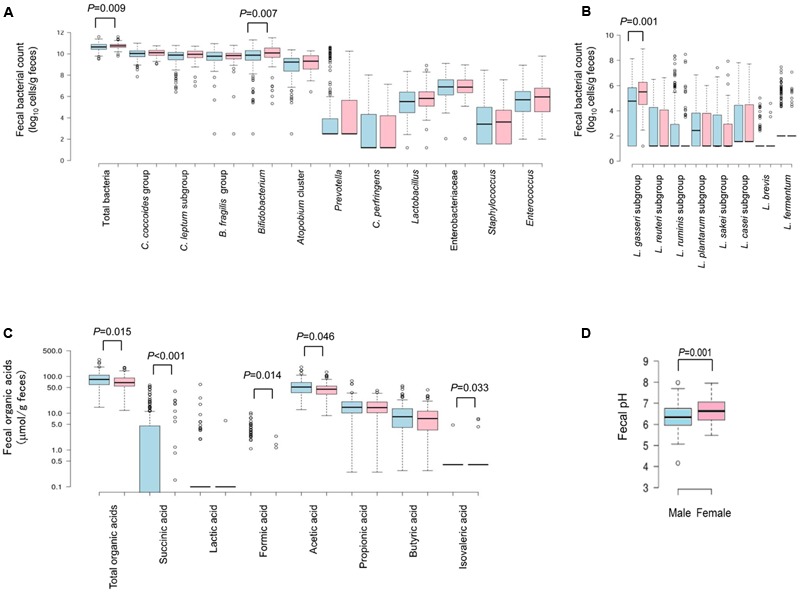
**Fecal count of major intestinal bacterial groups (A)** and Lactobacillus subgroups/species **(B)**, fecal concentration of organic acids **(C)**, and fecal pH **(D)** in healthy Japanese college students profiled by gender. Blue box: males (*n* = 212); pink box: females (*n* = 81). Center lines show the medians; box limits indicate the 25th and 75th percentiles as determined by R software; whiskers extend 1.5 times of the interquartile range from 25th and 75th percentiles; outliers are represented by dots. Only *P* < 0.05 is indicated here.

The fecal bacterial counts and organic acid concentrations categorized by host gender are shown in **Figure 1**. Compared to male counterparts, female subjects had significantly higher fecal count of total bacteria (*P* = 0.009), *Bifidobacterium* (*P* = 0.007) and *L. gasseri* subgroup (*P* = 0.001), higher fecal pH (*P* = 0.001), and lower fecal concentrations of total organic acids (*P* = 0.015), succinic acid (*P* < 0.001), formic acid (*P* = 0.014), acetic acid (*P* = 0.046) and isovaleric acid (*P* = 0.033).

### Fecal Microbiota and Organic Acids in Relation to the Frequency of Yogurt Consumption

The frequency of yogurt consumption associated positively with *Lactobacillus* (*P* = 0.001) and *Staphylococcus* (*P* = 0.021) fecal counts (**Table 3**). For *Lactobacillus*, the relationship was consistently significant in both male (*P* = 0.037) and female (*P* = 0.009) subjects (Supplementary Table 2). In contrast, the relationship with *Staphylococcus* was significant only in male subjects (*P* = 0.013) (Supplementary Tables 2A,B). When subdivided by gender, the count of Enterobacteriaceae was found to be negatively correlated (*P* = 0.045) with the frequency of yogurt consumption (Supplementary Table 2A).

**Table 3:** Fecal microbiota, organic acids and pH in relation to the frequency of yogurt consumption in healthy Japanese young adults (*n* = 293).

	Frequency (d/wk)				*P*
	6–7	3–5	1–2	<1	
Microbiota (log_10_ cells/g feces)					
Total bacteria	10.7 ± 0.4	10.6 ± 0.3	10.7 ± 0.4	10.6 ± 0.3	0.396
*C. coccoides* group	10.0 ± 0.4	10.0 ± 0.4	10.0 ± 0.5	10.0 ± 0.4	0.647
*C. leptum* subgroup	9.8 ± 0.6	9.8 ± 0.6	9.7 ± 0.8	9.7 ± 0.7	0.142
*B. fragilis* group	9.5 ± 1.3	9.8 ± 0.5	9.6 ± 1.3	9.7 ± 0.7	0.413
*Bifidobacterium*	9.5 ± 1.7	9.9 ± 0.6	9.7 ± 1.3	9.1 ± 2.1	0.060
*Atopobium* cluster	8.9 ± 0.9	9.2 ± 0.6	8.9 ± 1.4	8.8 ± 1.2	0.319
*Prevotella*	4.8 ± 3.0	3.6 ± 2.4	4.2 ± 2.7	3.8 ± 2.5	0.210
*C. perfringens*	2.8 ± 2.2	2.5 ± 1.8	2.8 ± 2.0	2.6 ± 2.0	0.865
*Lactobacillus*	6.2 ± 1.0	5.5 ± 1.5	5.3 ± 1.6	5.2 ± 1.8	0.001**
*L. gasseri* subgroup	5.7 ± 1.4	4.8 ± 1.8	4.2 ± 2.1	4.0 ± 2.1	<0.001**
*L. reuteri* subgroup	3.0 ± 1.8	2.6 ± 1.7	2.7 ± 1.7	2.5 ± 1.6	0.174
*L. ruminis* subgroup	2.1 ± 2.1	2.0 ± 1.8	2.2 ± 1.8	2.2 ± 1.9	0.269
*L. plantarum* subgroup	2.6 ± 1.6	2.4 ± 1.6	2.5 ± 1.6	2.6 ± 1.5	0.802
*L. sakei* subgroup	1.7 ± 1.0	1.9 ± 1.3	2.5 ± 1.7	2.5 ± 1.6	<0.001**
*L. casei* subgroup	3.0 ± 1.8	3.1 ± 2.0	2.8 ± 1.8	2.6 ± 1.8	0.005**
*L. brevis*	1.3 ± 0.5	1.3 ± 0.3	1.5 ± 1.0	1.4 ± 0.6	0.592
*L. fermentum*	2.4 ± 1.0	2.8 ± 1.5	2.6 ± 1.4	2.7 ± 1.5	0.655
Enterobacteriaceae	6.7 ± 1.5	6.5 ± 1.6	6.2 ± 1.8	6.8 ± 1.5	0.193
*Staphylococcus*	2.9 ± 1.7	3.5 ± 1.9	3.0 ± 1.7	3.8 ± 2.1	0.021**
*Enterococcus*	5.1 ± 2.0	5.6 ± 1.7	5.5 ± 1.8	5.3 ± 2.0	0.618
Organic acids (μmol/g feces)					
Total organic acids	82.2 ± 33.7	82.1 ± 50.3	89.4 ± 36.8	79.9 ± 35.3	0.786
Succinic acid	5.9 ± 11.6	1.6 ± 3.9	4.2 ± 9.8	4.0 ± 10.0	0.422
Lactic acid	0.1 ± 0.0	0.3 ± 1.0	0.9 ± 3.8	0.8 ± 5.7	0.247
Formic acid	0.2 ± 0.7	0.4 ± 1.3	0.5 ± 1.2	0.5 ± 1.7	0.601
Acetic acid	50.7 ± 21.0	53.4 ± 32.2	57.3 ± 24.0	50.5 ± 23.9	0.683
Propionic acid	16.0 ± 7.8	17.3 ± 12.9	16.9 ± 9.3	14.6 ± 7.6	0.218
Butyric acid	9.5 ± 7.5	9.2 ± 8.6	9.6 ± 6.7	9.5 ± 9.3	0.831
Isovaleric acid	0.4 ± 0.0	0.5 ± 0.5	0.5 ± 0.5	0.5 ± 0.8	0.550
Fecal pH	6.6 ± 0.5	6.5 ± 0.7	6.4 ± 0.6	6.4 ± 0.6	0.177

*^∗^*P* < 0.05, ^∗∗^*P* < 0.01, Jonckheere–Terpstra’s trend test.*

Among lactobacilli, *L. gasseri* subgroup (*P* < 0.001) and *L casei* subgroup (*P* = 0.005) counts had a positive association with the frequency of yogurt consumption, whereas *L. sakei* subgroup showed an inverse association (*P* < 0.001) with yogurt (**Table 3**). While the association with *L. gasseri* subgroup was consistent in both male (*P* = 0.007) and female (*P* < 0.001) subjects, *L. casei* and *L. sakei* subgroups showed significant correlation only in female (*P* < 0.001) and male (*P* < 0.001) subjects, respectively. The results of the trend test are elaborated in Supplementary Tables 2A,B.

### Gender-Stratified Correlation between Fecal Microbiota and the Frequency of yogurt Consumption

**FIGURE 2:**
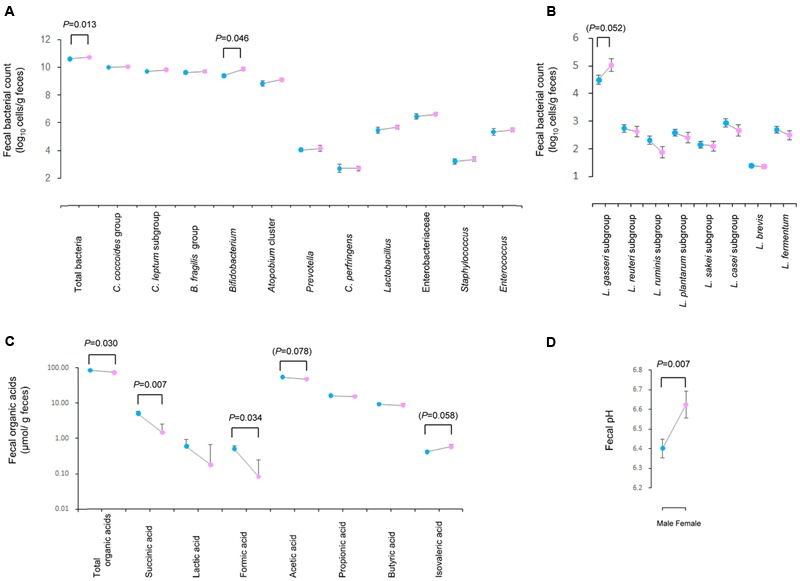
**Estimated marginal means calculated for fecal count of major intestinal bacterial groups (A)** and *Lactobacillus* subgroups/species **(B)**, fecal concentration of organic acids **(C)**, and fecal pH **(D)** adjusted for the frequency of yogurt consumption. Dots and error bas represent the marginal estimated mean and standard errors, respectively. Blue and pink dots represent male (n = 212) and female (n = 81) data. *P****-***value indicates the level of significance as tested by generalized linear model controlled for the frequency of yogurt consumption. In addition to *P* < 0.05, *P* < 0.1 is also presented in parenthesis.

Because the gender influence was expected (and also confirmed as described in the next section), the gender-dependent differences in microbiota and organic acids were also further analyzed by adjusting for yogurt consumption (**Figure 2**). Briefly, significant differences were confirmed in total bacteria (*P* = 0.013), *Bifidobacterium* (*P* = 0.046), fecal pH (*P* = 0.007), total organic acids (*P* = 0.030), succinic acid (*P* = 0.007) and formic acid (*P* = 0.034), along with insignificant differences in *L. gasseri* subgroup (*P* = 0.052), acetic acid (*P* = 0.078) and isovaleric acid (*P* = 0.058) (Supplementary Table 3).

**FIGURE 3:**
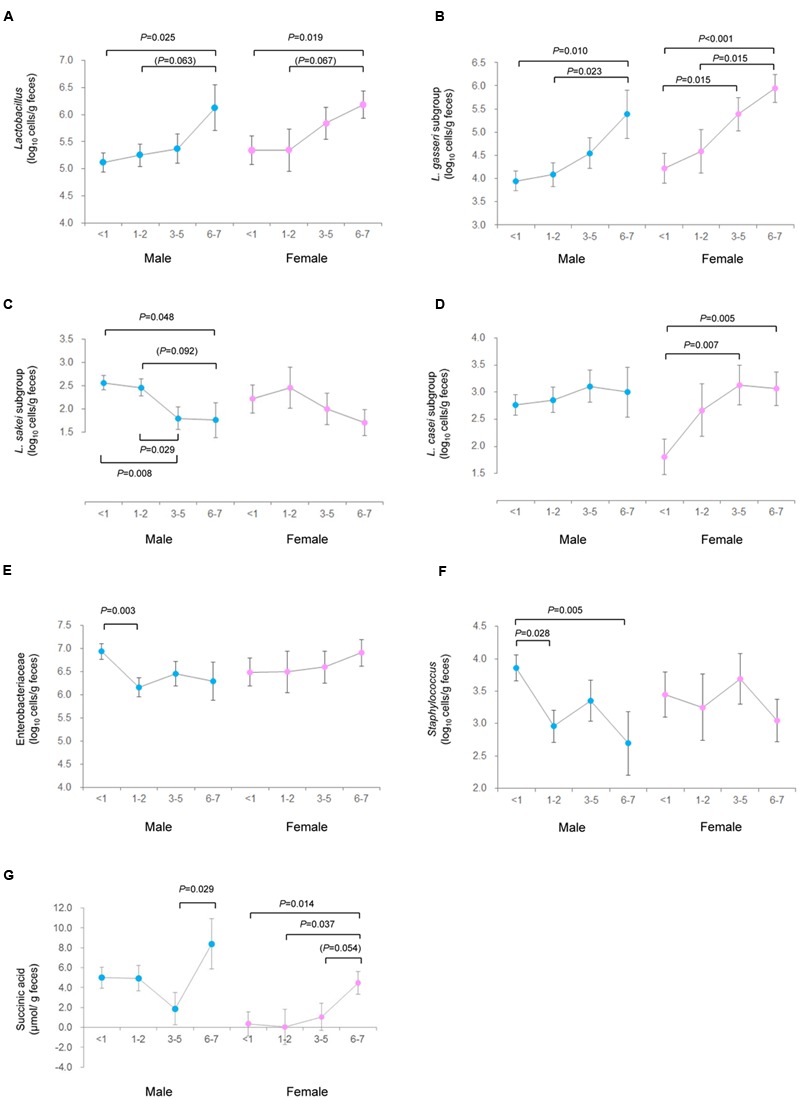
**Estimated marginal means of fecal bacterial count and organic acids concentration in male vs. female subjects profiled according to the frequency of yogurt consumption. (A)** Lactobacillus; **(B)** L. gasseri subgroup; **(C)** L. sakei subgroup; **(D)** *L. casei* subgroup; **(E)** Enterobacteriaceae; **(F)** Staphylococcus; and **(G)** succinic acid. Variables showing significant linear association with yogurt consumption in male or female subjects are presented. Dots and error bas represent the marginal estimated mean and standard errors, respectively. Blue and pink dots represent male and female data. *P*-value indicates the significance tested by generalized linear model. In addition to *P* < 0.05, *P* < 0.1 is also presented in parenthesis.

The GLM-based assessment of linear relation between microbiota and yogurt consumption frequency demonstrated positive associations for *Lactobacillus* and *L. gasseri* subgroup in both male and female subjects. However, several gender-specific disparities in this association were also noted. In male subjects, Enterobacteriaceae, *Staphylococcus* and *L. sakei* subgroup correlated negatively with yogurt consumption; whereas in females, *L. casei* subgroup and succinic acid shared a positive association with yogurt consumption (**Table 4**; the entire results are summarized in Supplementary Table 4). Based on these variables that confirmed the linear association in male and/or female subjects, marginal estimated means for each unit of yogurt-eating frequency (i.e., <1, 1–2, 3–5, and 6–7 d/wk) were also calculated and compared (**Figure 3**) which revealed a similar trend as observed above for most of the variables in males as well as in females, thereby confirming the influence of yogurt consumption on these variables.

**Table 4:** Linearity between the frequency of yogurt consumption and the fecal microbiota and organic acids, as analyzed by generalized linear model.

Linearity	Male (*n* = 212)			Female (*n* = 81)		
	Coefficient	*SE*	*P*	Coefficient	*SE*	*P*
Fecal bacterial count (log_10_ cells/g feces)		
*Lactobacillus*	0.238	0.121	0.049*	0.293	0.117	0.012*
*L. gasseri* subgroup	0.389	0.148	0.009**	0.587	0.142	<0.001**
*L. sakei* subgroup	-0.311	0.108	0.004**	-0.184	0.134	0.169
*L. casei* subgroup	0.122	0.134	0.363	0.425	0.147	0.004**
Enterobacteriaceae	-0.245	0.119	0.039*	0.136	0.135	0.317
*Staphylococcus*	-0.345	0.142	0.015*	-0.097	0.156	0.534
Organic acids (μmol/g feces)		
Succinic acid	-0.108	0.733	0.883	1.331	0.544	0.014*

*Frequency of yogurt coded as 1: <1; 2: 1–2; 3: 3–5; and 4: 5–6 (d/wk). SE, standard error, 95% CI; 95% confidential interval, ^∗^*P* < 0.05, ^∗∗^*P* < 0.01. Only bacteria and organic acids with significant correlation are shown here.*

In contrast, when adjusted for gender, the levels in <1 d/wk category were found to be significant in case of *L. casei* subgroup (*P* = 0.020) and succinic acid (*P* = 0.027). Among variables that otherwise did not show a linear association with yogurt frequency, significant gender differences were seen in total bacteria (*P* = 0.021), *C. coccoides* group (*P* = 0.035), *Bifidobacterium* (*P* = 0.041), *Enterococcus* (*P* = 0.036), isovaleric acid (*P* < 0.001), and pH (*P* < 0.001) in subjects taking yogurt less than once in a week (males *n* = 93, females *n* = 24) (Supplementary Table 5).

## Discussion

It is not uncommon for college students to participate in gut microbiota-related studies; however, to our knowledge, studies focusing specifically on the diet–microbiota associations in graduate students have not yet been performed. Furthermore, human gender-microbiota association has also not been assessed thoroughly in cohorts having homogeneous ethnic background, age, lifestyle etc. As such, the gut microbiota of collegiate students are of particular interest in that they share quite similar lifestyle, age, residential area, environment, etc. thereby providing an ideal cohort for such explorations. Therefore, with an aim to understand the association of gut microbiota with dietary habits (specifically yogurt consumption) and host gender in healthy young adults, we herein investigated the fecal microbiota composition of collegiate freshmen (first-year graduate students) nearly 3 months after they had moved into the student dormitory. This 3-month window was included to ensure that the students (as well as their gut microbiota) are settled according to the new lifestyle, because factors such as relocating to a new place and adapting to new routines, activities, etc. may have some stress on the freshmen and might also influence their gut microbiota populations.

Recent studies using comprehensive metagenomic analysis of bacterial 16S rRNA gene sequences have been remarkable in unraveling the overall configuration of the human intestinal microbiota (Mueller et al., 2006; Robles-Alonso and Guarner, 2014). However, these analyses do not provide adequate and quantitative information about the actual population levels of the subdominant bacterial inhabitants (e.g., facultative anaerobes including staphylococci, enterococci, lactobacilli; and opportunistic pathogens such as *C. perfringens*) that are generally present in low levels (e.g., <10^4-5^ cells/g feces) but may otherwise be a regular and important component of the adult gut microbiota. From this perspective, the RT-qPCR assays used in the present study targets bacterial rRNA molecules thereby providing a detection sensitivity that is approximately 100–1000 fold higher as compared with other molecular biological methods (Matsuda et al., 2007, 2009; Kubota et al., 2010; Kurakawa et al., 2012). Therefore, with an aim to encompass a wide range of both predominant and subdominant gut bacterial groups, we specifically employed RT-qPCR approach for the present investigation.

Overall, the gut microbiota of first-year graduate students sampled in this study were found to be dominated by *C. coccoides* group, *C. leptum* subgroup, *B. fragilis* group, *Bifidobacterium*, and *Atopobium* cluster. This was expected and concurs well with previous reports (Matsuki et al., 2004; Matsuda et al., 2007; Nakayama et al., 2015); however the gender-specific differences in the microbiota response to dietary habits were rather fortuitous and intriguing.

Females had a higher abundance of total bacteria, *Bifidobacterium* and *L. gasseri* subgroup in the fecal microbiota, and these differences might plausibly directly or indirectly underlie the lower concentrations of short-chain fatty acids including succinic acid, formic acid and isovaleric acid and accordingly the higher fecal pH observed in female subjects. In addition, the yogurt-microbiota correlation was also found to differ significantly according to the host gender: yogurt consumption associated negatively with *L. sakei* subgroup, Enterobacteriaceae and *Staphylocossus* counts in males whilst sharing a positive association with *L. casei* subgroup count and succinic acid concentration in female subjects. Furthermore, among subjects consuming yogurt less than once per week, females had a significant higher *C. coccoides* group counts. Such gender-specific differences in the microbiota composition as well as in its response to yogurt consumption, that otherwise remain undemonstrated thus far particularly in cohorts homogeneous for age, lifestyle, cultural background, etc., would certainly be a new and intriguing theme for prospective studies on human intestinal microbiota.

Whether and how gender influences the gut microbiota still remains unclear, although a few studies do have suggested gender-specific variations in human intestinal microbiota (Mueller et al., 2006) and its response to diet (Bolnick et al., 2014). Markle et al. (2013) showed that these differences could depend on the stages of host sexual maturation. With an age range of 18–23 years old, our subjects can be regarded as sexually mature and hence the gender differences in their gut microbiota could be considered established and extrapolatable to human adults in general, at least in Japanese population. Notably, the higher abundance of *Bifidobacterium* and *L. gasseri* subgroup in female (vs. male) students was found to be one of the major gender-specific differences in intestinal microbiota composition. *Lactobacillus* community, including *L. gasseri* subgroup, predominates the human vaginal microbiota worldwide (Lamont et al., 2011; Ravel et al., 2011; Kurakawa et al., 2015b). Furthermore, the ingestion of exogenous *Lactobacillus* is also known to up-regulate the intestinal carriage of *Bifidobacterium* and *Lactobacillus* (Wang et al., 2015; Nagata et al., 2016). Therefore, it may be reasonable to hypothesize that vaginal lactobacilli, if translocated to gut, might increase the intestinal *Bifidobacterium* and *Lactobacillus* carriage. Maybe this mechanism was underlying the gender differences in fecal *Bifidobacterium* and *Lactobacillus* counts observed in this study. However, such mechanism of the transmission of vaginal lactobacilli to the intestine, possibly via circulation or translocation from vagina to anal/perianal niche, remains unidentified. Further studies are warranted to explore and clarify these mechanisms so as to configure the gender-specific differences in the human intestinal microbiota.

In our previous studies, we have demonstrated that the consumption of a probiotic yogurt drink (fermented with *L. casei* strain Shirota) increases total fecal *Lactobacillus* count and decreases Enterobacteriaceae and *Staphylococcus* counts (Tsuji et al., 2014; Wang et al., 2015). In addition, elevations in fecal *Bifidobacterium* and *Lactobacillus* carriage have also been observed following the consumption of yogurt fermented with *Bifidobacterium lactis* (Ahmed et al., 2007; Savard et al., 2011) and *Lactobacillus fermentum* (West et al., 2011), respectively. In the present study also, yogurt consumption was found to be associated with increase in *Lactobacillus*, especially *L. gasseri* subgroup, and decrease in Enterobacteriaceae and *Staphylococcus* (male only) counts, thereby corroborating that the consumption of yogurt and/or probiotics could enhance the intestinal population level of *Lactobacillus* while reducing that of Enterobacteriaceae and *Staphylococcus*.

Several previous cross-sectional studies on people having broad backgrounds have demonstrated significant association of microbiota with dietary elements such as fat, carbohydrates (Wu et al., 2011), vegetarian diet (Zimmer et al., 2012), and an agrarian diet (which is rich in fruits and legume fiber) (De Filippo et al., 2010). The influence of plant-based diet (David et al., 2014) as well as the dietary fiber (Simpson and Campbell, 2015) on human gut microbiota has also been recently highlighted. Such diet–microbiota associations have also been reported in varying geographical backgrounds, e.g. subjects from rural Africa or Europe (De Filippo et al., 2010) or other different countries (Lozupone et al., 2012; Yatsunenko et al., 2012; Nakayama et al., 2015) or from different socioeconomic groups (Benno et al., 1989; Mello et al., 2009; Claesson et al., 2012). These studies consistently suggested the diet–microbiota interaction and also its consequent influence on fecal short chain fatty acid concentrations. Fecal acetic and propionic acids are well known to confer various beneficial effects on the host, such as provision of energy (Kasubuchi et al., 2015), protection from infections (Fukuda et al., 2011), and positive modulation of the metabolism (Kasubuchi et al., 2015; Sonnenburg and Bäckhed, 2016). In these contexts, and also given a gradual decrease in average vegetable intake among contemporary younger generation in Japan (Ministry of Health Labour and Welfare, 2007), we speculate that such dietary habits, e.g., differences in the intake of fruits, vegetables, fiber, gluten, meat, and eggs etc. might also have confounded our results to some extent but these influences were difficult to scrutinize in this highly homogenous cohort. Nevertheless, our data do provide new and valuable information on the diet–microbiota association in the absence of age, lifestyle, ethnic and environmental diversities and how this relationship could be influenced by the host gender, thereby underscoring the need for further elucidation of the influence of dietary habits and factors on gut microbiota especially by considering gender as an important component.

There are some limitations in this study. First, as described above, the diet–microbiota association might also have been confounded by some unknown (i.e., herein unstudied) elements such as history of major illnesses and/or drug use, early life environmental and microbiota factors, upbringing and household/family environment, physical activities, miscellaneous dietary habits etc. Differences in the bacterial strains used in different yogurt products might also influence the host microbiota but this information could not be obtained for this cohort. Second, as mentioned before, the fecal microbiota were analyzed by using RT-qPCR assays which allow a sensitive quantification of the majority of predominant human gut bacterial clades (Shiori et al., 2006) and clinically important pathobionts and opportunistic pathogens; but these bacteria might not represent the entire gut microbiota. Hence, it will be interesting to see how these findings are corroborated in further studies using all-inclusive analytical methods. Nevertheless, given that most of the studies employ DNA-based PCR or sequencing methods to assess the microbiota, our RT-qPCR data (which is comparatively highly sensitive and correspond to viable cell counts) (Matsuda et al., 2009) could prove to be unique and informative and hence should fortify the existing literature of human gut microbiota with important numerical information about the intestinal carriage of dominant gut bacterial groups.

## Conclusion

We herein demonstrate the features of the intestinal microbiota in healthy Japanese young adults sharing same ethnicity, age, and life circumstances and routines. The data show how the composition of the adult human gut microbiota could differ in relation to host gender as well as dietary habits such as the frequency of yogurt consumption, while at the same time revealing considerable gender-specific disparities in the yogurt-microbiota correlation. The findings are hypothesis-generating and continue to reveal the complex dynamics of the human intestinal microbiota, and hence should prove to be informative and important for prospective studies exploring the intricate triangle of diet, microbiota and health.

## Author Contributions

YS, KI, KaS, KeS, KN, CW, SN, and YY: designed the study; YS, KI, KaS, SK, and KeS: coordinated questionnaire and sample collection; TA, TT, HT, and KN: performed experiments; YS: analyzed data; YS and HT: interpreted data; YS: drafted manuscript; HT and RN: revised and edited manuscript; YS, KI, KaS, SK, KeS, TA, TT, HT, KN, RN, CW, SN, and YY: checked and approved final version of manuscript.

## Conflict of Interest Statement

TA, HT, TT, KN are employees of Yakult Honsha Co. Ltd., Japan. SN and YY have received unrestricted research grants from Yakult Honsha Co. Ltd. The funding agency had no role in study design, data collection and analysis, decision to publish, or preparation of the manuscript. The other authors declare that the research was conducted in the absence of any commercial or financial relationships that could be construed as a potential conflict of interest.
