# Supplementary material for: Association between Yogurt Consumption and Intestinal Microbiota in Healthy Young Adults Differs by Host Gender
Source: Front Microbiol. 2017 May 11;8:847. doi: 10.3389/fmicb.2017.00847 (PMC5425481; doi:10.3389/fmicb.2017.00847)
Supplement: Supplementary file 1 [file Table_1.PDF]

**Supplementary Table 1. Frequency of yoghurt consumption during 2 months before start of the study.**

| | | Frequency (d/wk) | | | | P | |
|---|---|---|---|---|---|---|---|
| | | 6–7 | 3–5 | 1–2 | <1 | | |
| Total | n | 43 | 58 | 75 | 117 | | |
| | % | 15% | 20% | 26% | 40% | | |
| Male | n | 16 | 39 | 64 | 93 | <0.001 | ** |
| | % | 8% | 18% | 30% | 44% | | |
| Female | n | 27 | 19 | 11 | 24 | | |
| | % | 33% | 23% | 14% | 30% | | |

**P<0.01 (mean difference between male and female subjects by Mann–Whitney's U test)
