# Supplementary material for: Association between Yogurt Consumption and Intestinal Microbiota in Healthy Young Adults Differs by Host Gender
Source: Front Microbiol. 2017 May 11;8:847. doi: 10.3389/fmicb.2017.00847 (PMC5425481; doi:10.3389/fmicb.2017.00847)
Supplement: Supplementary file 2 [file Table_2.PDF]

Supplementary Table 2. Fecal microbiota composition in relation to the frequency of yoghurt consumption. (A) Total subjects. **(B)** Male. **(C)** Female.
*P<0.05, **P<0.01 (Jonckheere-Terpstra test)

A Male

Frequency (d/wk)	6-7			3-5			1-2			<1				
	n	mean	s.d	n	mean	s.d	n	mean	s.d	n	mean	s.d	P	
Microbiota (log_{10} cells/g feces)														
Total bacteria	16	10.60	0.41	39	10.65	0.35	64	10.62	0.42	93	10.60	0.32	0.471	
C. coccoides group	16	10.00	0.51	39	10.11	0.36	64	9.98	0.46	93	9.96	0.44	0.181	
C. leptum subgroup	16	9.72	0.74	39	9.87	0.61	64	9.60	0.84	93	9.68	0.69	0.273	
B. fragilis group	16	9.47	1.16	39	9.85	0.39	64	9.49	1.40	93	9.71	0.61	0.847	
Bifidobacterium	16	9.17	2.20	39	9.82	0.68	64	9.66	1.37	93	8.99	2.24	0.195	
Atopobium cluster	16	8.69	1.15	39	9.12	0.59	64	8.88	1.44	93	8.71	1.32	0.274	
Prevotella	16	4.79	3.23	39	3.46	2.41	64	4.32	2.87	93	3.61	2.35	0.440	
C. perfringens	16	2.80	2.57	39	2.72	1.86	64	2.68	1.89	93	2.60	2.02	0.653	
Lactobacillus	16	6.12	0.97	39	5.37	1.74	64	5.25	1.72	93	5.11	1.75	0.037	*
L. gasseri subgroup	16	5.39	1.86	39	4.55	1.96	64	4.08	2.18	93	3.94	2.07	0.007	**
L. reuteri subgroup	16	3.20	1.79	39	2.51	1.73	64	2.72	1.75	93	2.51	1.63	0.321	
L. ruminis subgroup	16	2.52	2.37	39	2.24	1.99	64	2.30	1.87	93	2.14	1.77	0.772	
L. plantarum subgroup	16	2.55	1.76	39	2.52	1.51	64	2.53	1.63	93	2.70	1.51	0.377	
L. sakei subgroup	16	1.76	1.08	39	1.80	1.24	64	2.46	1.77	93	2.56	1.47	0.000	**
L. casei subgroup	16	3.00	1.64	39	3.11	2.06	64	2.86	1.81	93	2.76	1.89	0.094	
L. brevis	16	1.37	0.45	39	1.28	0.36	64	1.51	0.94	93	1.40	0.65	0.769	
L. fermentum	16	2.60	1.32	39	2.80	1.49	64	2.64	1.37	93	2.72	1.55	0.938	
Enterobacteriaceae	16	6.30	1.84	39	6.46	1.76	64	6.16	1.86	93	6.94	1.40	0.045	*
Staphylococcus	16	2.70	1.60	39	3.35	1.95	64	2.96	1.66	93	3.86	2.22	0.013	*
Enterococcus	16	5.21	1.75	39	5.57	1.86	64	5.52	1.84	93	5.07	1.91	0.114	
Organic acids (μ mol/g feces)														
Total organic acid	16	80.68	30.03	39	86.11	53.60	64	93.85	35.98	93	81.49	36.13	0.565	
succinic acid	16	8.39	13.90	39	1.86	4.20	64	4.94	10.46	93	4.99	10.97	0.624	
lactic acid	16	0.10	0.00	39	0.24	0.65	64	1.04	4.05	93	1.02	6.34	0.380	
formic acid	16	0.32	1.17	39	0.48	1.52	64	0.61	1.33	93	0.63	1.90	0.817	
acetic acid	16	49.69	20.10	39	55.96	33.43	64	59.80	23.48	93	50.93	24.63	0.303	
propionic acid	16	14.26	7.37	39	18.43	14.30	64	17.48	9.50	93	14.31	7.56	0.153	
butyric acid	16	8.10	5.99	39	9.32	9.03	64	10.04	7.00	93	9.78	9.30	0.822	
isovaleric acid	16	0.40	0.00	39	0.40	0.00	64	0.47	0.55	93	0.40	0.00	0.741	
pH	16	6.52	0.47	39	6.44	0.69	63	6.31	0.59	93	6.33	0.55	0.176	

Jonckheere-Terpstra test

B Female

Frequency (d/wk)	6-7			3-5			1-2			<1				
	n	mean	s.d	n	mean	s.d	n	mean	s.d	n	mean	s.d	P	
Microbiota (log_{10} cells/g feces)														
Total bacteria	27	10.75	0.33	19	10.59	0.34	11	10.84	0.29	24	10.79	0.31	0.583	
C. coccoides group	27	10.06	0.33	19	9.90	0.37	11	10.09	0.45	24	10.16	0.30	0.225	
C. leptum subgroup	27	9.90	0.50	19	9.71	0.72	11	10.01	0.52	24	9.72	0.81	0.649	
B. fragilis group	27	9.49	1.45	19	9.57	0.53	11	10.00	0.28	24	9.78	0.88	0.059	
Bifidobacterium	27	9.76	1.38	19	10.07	0.42	11	9.94	1.16	24	9.76	1.35	0.686	
Atopobium cluster	27	9.08	0.70	19	9.32	0.61	11	8.88	1.15	24	9.20	0.77	0.548	
Prevotella	27	4.88	2.87	19	3.78	2.39	11	3.30	1.38	24	4.57	2.83	0.556	
C. perfringens	27	2.76	2.02	19	1.99	1.65	11	3.40	2.31	24	2.67	2.09	0.906	
Lactobacillus	27	6.19	0.97	19	5.84	0.82	11	5.35	0.95	24	5.34	1.95	0.009	**
L. gasseri subgroup	27	5.94	0.92	19	5.39	1.34	11	4.58	1.48	24	4.22	2.30	0.000	**
L. reuteri subgroup	27	2.83	1.79	19	2.64	1.58	11	2.67	1.83	24	2.35	1.71	0.307	
L. ruminis subgroup	27	1.89	1.87	19	1.57	1.18	11	1.43	0.76	24	2.59	2.18	0.119	
L. plantarum subgroup	27	2.67	1.53	19	2.25	1.70	11	2.59	1.40	24	2.08	1.39	0.193	
L. sakei subgroup	27	1.71	1.00	19	2.01	1.49	11	2.46	1.33	24	2.22	1.99	0.203	
L. casei subgroup	27	3.06	1.94	19	3.13	1.77	11	2.67	1.97	24	1.80	0.82	0.005	**
L. brevis	27	1.30	0.52	19	1.20	0.00	11	1.72	1.18	24	1.20	0.00	0.896	
L. fermentum	27	2.23	0.84	19	2.73	1.52	11	2.58	1.31	24	2.45	1.22	0.524	
Enterobacteriaceae	27	6.91	1.20	19	6.60	1.25	11	6.50	1.66	24	6.49	1.93	0.607	
Staphylococcus	27	3.05	1.82	19	3.69	1.78	11	3.25	1.76	24	3.44	1.63	0.429	
Enterococcus	27	4.98	2.17	19	5.68	1.49	11	5.35	1.97	24	5.98	2.15	0.073	
Organic acids (μ mol/g feces)														
Total organic acid	27	83.04	36.26	19	73.79	43.04	11	63.52	31.72	24	73.81	32.09	0.407	
succinic acid	27	4.46	10.07	19	1.04	3.05	11	0.04	0.00	24	0.36	1.22	0.264	
lactic acid	27	0.10	0.00	19	0.43	1.42	11	0.10	0.00	24	0.10	0.00	0.859	
formic acid	27	0.08	0.27	19	0.21	0.58	11	0.03	0.00	24	0.03	0.00	0.362	
acetic acid	27	51.27	21.80	19	48.11	29.50	11	42.85	22.99	24	48.60	21.44	0.637	
propionic acid	27	16.99	7.94	19	14.94	9.32	11	13.50	7.56	24	15.94	7.84	0.595	
butyric acid	27	10.31	8.33	19	9.03	7.85	11	7.20	4.45	24	8.38	9.24	0.214	
isovaleric acid	27	0.40	0.00	19	0.60	0.89	11	0.40	0.00	24	0.94	1.84	0.160	
pH	27	6.61	0.59	19	6.57	0.65	11	6.60	0.50	24	6.72	0.43	0.416	

Jonckheere-Terpstra test
