# Supplementary material for: Association between Yogurt Consumption and Intestinal Microbiota in Healthy Young Adults Differs by Host Gender
Source: Front Microbiol. 2017 May 11;8:847. doi: 10.3389/fmicb.2017.00847 (PMC5425481; doi:10.3389/fmicb.2017.00847)
Supplement: Supplementary file 3 [file Table_3.PDF]

Supplementary Table 3. Estimated marginal means and standard errors of fecal counts of various bacterial groups and fecal concentration of organic acids controlled for frequency of yoghurt consumption in healthy young adults enrolled in the study.

	Male		Female		P	
	mean	s.e.	mean	s.e.		
Total bacteria	10.62	0.03	10.74	0.04	0.013	*
C. coccoides group	10.01	0.03	10.05	0.05	0.510	
C. leptum subgroup	9.72	0.06	9.83	0.08	0.249	
B. fragilis group	9.63	0.08	9.71	0.11	0.552	
Bifidobacterium	9.41	0.14	9.88	0.19	0.046	*
Atopobium cluster	8.85	0.10	9.12	0.13	0.098	
Prevotella	4.04	0.22	4.13	0.30	0.811	
C. perfringens	2.70	0.17	2.71	0.23	0.981	
Lactobacillus	5.46	0.13	5.68	0.19	0.350	
L. gasseri subgroup	4.49	0.16	5.03	0.23	0.052	
L. reuteri subgroup	2.74	0.14	2.62	0.20	0.645	
L. ruminis subgroup	2.30	0.15	1.87	0.22	0.105	
L. plantarum subgroup	2.58	0.13	2.40	0.18	0.430	
L. sakei subgroup	2.15	0.13	2.10	0.18	0.811	
L. casei subgroup	2.93	0.15	2.67	0.21	0.305	
L. brevis	1.39	0.05	1.35	0.08	0.703	
L. fermentum	2.69	0.12	2.50	0.16	0.338	
Enterobacteriaceae	6.47	0.13	6.62	0.19	0.495	
Staphylococcus	3.22	0.16	3.36	0.22	0.604	
Enterococcus	5.34	0.16	5.50	0.22	0.573	
Total organic acids	85.53	3.22	73.54	4.49	0.030	*
succinic acid	5.04	0.77	1.48	1.07	0.007	**
lactic acid	0.60	0.34	0.18	0.48	0.476	
formic acid	0.51	0.12	0.08	0.16	0.034	*
acetic acid	54.09	2.11	47.71	2.95	0.078	
propionic acid	16.12	0.78	15.34	1.08	0.560	
butyric acid	9.31	0.69	8.73	0.97	0.625	
isovaleric acid	0.42	0.05	0.59	0.07	0.058	
pH	6.40	0.05	6.62	0.07	0.007	**

*P<0.05, **P<0.01
