# Supplementary material for: Association between Yogurt Consumption and Intestinal Microbiota in Healthy Young Adults Differs by Host Gender
Source: Front Microbiol. 2017 May 11;8:847. doi: 10.3389/fmicb.2017.00847 (PMC5425481; doi:10.3389/fmicb.2017.00847)
Supplement: Supplementary file 4 [file Table_4.PDF]

Supplementary Table 4. Linearity of fecal microbiota and organic acids with the frequency of yoghurt consumption, as analyzed by generalized linear model. Frequency of yogurt coded as 1; <1, 2; 1–2, 3; 3–5, and 4; 5–6 (d/wk).

Linerarity	Male						Female					
	C	SE	95% CI		P		C	SE	95% CI		P	
			Lower	Upper					Lower	Upper		
Microbiota (log_{10} cells/g feces)												
Total bacteria	0.012	0.026	−0.039	0.063	0.650		−0.025	0.029	−0.083	0.032	0.388	
C. coccoides group	0.040	0.031	−0.022	0.101	0.206		−0.045	0.032	−0.107	0.016	0.150	
C. leptum subgroup	0.043	0.052	−0.059	0.145	0.410		0.040	0.059	−0.076	0.155	0.500	
B. fragilis group	−0.017	0.068	−0.149	0.116	0.806		−0.115	0.090	−0.292	0.061	0.201	
Bifidobacterium	0.237	0.129	−0.016	0.491	0.066		0.012	0.106	−0.195	0.219	0.912	
Atopobium cluster	0.092	0.089	−0.083	0.267	0.303		−0.011	0.070	−0.148	0.126	0.874	
Prevotella	0.195	0.187	−0.172	0.562	0.297		0.117	0.236	−0.345	0.579	0.619	
C. perfringens	0.062	0.142	−0.216	0.341	0.661		−0.053	0.181	−0.408	0.303	0.770	
Lactobacillus	0.238	0.121	0.001	0.475	0.049	*	0.293	0.117	0.064	0.521	0.012	*
L. gasseri subgroup	0.389	0.148	0.099	0.679	0.009	**	0.587	0.142	0.308	0.865	0.000	**
L. reuteri subgroup	0.129	0.121	−0.108	0.366	0.287		0.152	0.153	−0.148	0.451	0.321	
L. ruminis subgroup	0.096	0.135	−0.168	0.360	0.477		−0.216	0.157	−0.523	0.091	0.168	
L. plantarum subgroup	−0.072	0.112	−0.290	0.147	0.521		0.166	0.135	−0.098	0.430	0.219	
L. sakei subgroup	−0.311	0.108	−0.522	−0.100	0.004	**	−0.184	0.134	−0.447	0.079	0.169	
L. casei subgroup	0.122	0.134	−0.141	0.384	0.363		0.425	0.147	0.137	0.714	0.004	**
L. brevis	−0.029	0.050	−0.127	0.070	0.567		0.002	0.049	−0.093	0.098	0.962	
L. fermentum	−0.009	0.105	−0.214	0.196	0.931		−0.059	0.108	−0.270	0.152	0.581	
Enterobacteriaceae	−0.245	0.119	−0.477	−0.012	0.039	*	0.136	0.135	−0.130	0.401	0.317	
Staphylococcus	−0.345	0.142	−0.623	−0.067	0.015	*	−0.097	0.156	−0.403	0.209	0.534	
Enterococcus	0.149	0.134	−0.113	0.411	0.264		−0.294	0.178	−0.642	0.054	0.098	
Organic acids (μ mol/g feces)												
Total organic acids	1.311	2.836	−4.247	6.869	0.644		3.428	3.239	−2.920	9.775	0.290	
succinic acid	−0.108	0.733	−1.544	1.329	0.883		1.331	0.544	0.265	2.397	0.014	*
lactic acid	−0.331	0.340	−0.998	0.336	0.330		0.020	0.062	−0.101	0.142	0.745	
formic acid	−0.089	0.116	−0.317	0.138	0.440		0.027	0.029	−0.030	0.084	0.348	
acetic acid	1.207	1.861	−2.441	4.855	0.517		1.111	2.122	−3.049	5.271	0.601	
propionic acid	1.018	0.698	−0.350	2.386	0.145		0.398	0.733	−1.039	1.834	0.587	
butyric acid	−0.384	0.598	−1.557	0.789	0.521		0.705	0.721	−0.708	2.118	0.328	
isovaleric acid	0.002	0.022	−0.040	0.045	0.913		−0.159	0.097	−0.350	0.031	0.101	
pH	0.058	0.042	−0.024	0.140	0.164		−0.037	0.049	−0.133	0.058	0.442	

C: coefficient, SE; standard error, 95%CI; 95% confidential interval, *; P<0.05, **; P<0.01
