# Supplementary material for: Association between Yogurt Consumption and Intestinal Microbiota in Healthy Young Adults Differs by Host Gender
Source: Front Microbiol. 2017 May 11;8:847. doi: 10.3389/fmicb.2017.00847 (PMC5425481; doi:10.3389/fmicb.2017.00847)
Supplement: Supplementary file 5 [file Table_5.PDF]

Supplementary Table 5. Fecal microbiota composition and fecal organic acids in subjects consuming yoghurt less than once in a week, as assessed by generalized linear model.

	Male (n=93)		Female (n=24)			
	mean	s.e.	mean	s.e.	P	
Microbiota ($\log_{10}$ cells/g feces)						
Total bacteria	10.6	0.0	10.8	0.1	0.021	*
C. coccoides group	10.0	0.0	10.2	0.1	0.035	*
C. leptum subgroup	9.7	0.1	9.7	0.1	0.843	
B. fragilis group	9.7	0.1	9.8	0.2	0.729	
Bifidobacterium	9.0	0.2	9.8	0.3	0.041	*
Atopobium cluster	8.7	0.1	9.2	0.2	0.059	
Prevotella	3.6	0.3	4.6	0.5	0.103	
C. perfringens	2.6	0.2	2.7	0.4	0.881	
Lactobacillus	5.1	0.2	5.3	0.3	0.527	
L. gasseri subgroup	3.9	0.2	4.2	0.4	0.529	
L. reuteri subgroup	2.5	0.2	2.3	0.3	0.679	
L. ruminis subgroup	2.1	0.2	2.6	0.4	0.278	
L. plantarum subgroup	2.7	0.2	2.1	0.3	0.079	
L. sakei subgroup	2.6	0.2	2.2	0.3	0.309	
L. casei subgroup	2.8	0.2	1.8	0.4	0.020	*
L. brevis	1.4	0.1	1.2	0.1	0.175	
L. fermentum	2.7	0.1	2.4	0.3	0.385	
Enterobacteriaceae	6.9	0.2	6.5	0.3	0.219	
Staphylococcus	3.9	0.2	3.4	0.4	0.337	
Enterococcus	5.1	0.2	6.0	0.4	0.036	*
Organic acids (μmol/g feces)						
Total organic acids	81.5	4.0	73.8	7.8	0.379	
succinic acid	5.0	0.9	0.4	1.9	0.027	*
lactic acid	1.0	0.4	0.1	0.8	0.321	
formic acid	0.6	0.1	0.0	0.3	0.055	
acetic acid	50.9	2.6	48.6	5.1	0.685	
propionic acid	14.3	1.0	15.9	1.9	0.436	
butyric acid	9.8	0.9	8.4	1.7	0.456	
isovaleric acid	0.4	0.1	0.9	0.1	<0.001	**
pH	6.3	0.1	6.7	0.1	<0.001	**
